# Multiple primary melanoma in association with other personal and familial cancers

**DOI:** 10.1002/cam4.5088

**Published:** 2022-08-05

**Authors:** Xi Yang, Lilit Karapetyan, Ziyu Huang, Andrew D. Knight, Sneha Rajendran, Cindy Sander, Christopher P. Minnier, Melissa J. Wilson, John M. Kirkwood

**Affiliations:** ^1^ Department of Medicine, Division of General Internal Medicine University of Pittsburgh Medical Center Pittsburgh Pennsylvania USA; ^2^ Department of Medicine, Division of Hematology/Oncology; University of Pittsburgh Medical Center Hillman Cancer Center Pittsburgh Pennsylvania USA; ^3^ UPMC Hillman Cancer Center Biostatistics Facility Pittsburgh Pennsylvania USA; ^4^ UPMC Hillman Cancer Center Pittsburgh Pennsylvania USA; ^5^ Department of Medicine University of Pittsburgh Pittsburgh Pennsylvania USA

**Keywords:** first‐degree relatives, multiple primary melanoma, nonskin cancers, prostate cancer, single primary melanoma

## Abstract

**Background:**

Multiple primary melanoma (MPM) is known to be associated with familial melanoma. However, the association between MPM and other personal and familial cancers is not well documented. The objective of this study was to evaluate the association between MPM and personal history of other cancers or cancer history among first‐degree relatives (FDRs).

**Methods:**

We performed a retrospective case–control study including cases with gender‐matched MPM and single primary melanoma (SPM) at a 1:2 ratio from the University of Pittsburgh Cancer Institute Melanoma Center Biological Sample and Nevus Bank. The associations between MPM and other cancers were evaluated using univariable and multivariable logistic regression models.

**Results:**

In total, 378 patients (44.2% men; median age 52 years) were enrolled, including 252 with SPM and 126 with MPM. In comparison to patients with SPM, patients with MPM were more likely to have squamous cell carcinoma (odds ratio [OR] 1.95, 95% confidence interval [CI] 1.001–3.79, *p* = 0.047) and prostate cancer (OR 2.72, 95% CI 1.07–7.01, *p* = 0.034). FDRs of patients with MPM had higher prevalence of melanoma (OR 2.37, 95% CI 1.31–4.28, *p* = 0.004) and prostate cancer (OR 2.92, 95% CI 1.47–6.14, *p* = 0.002) but not other cancers. In multivariable analysis, the association remained significant between MPM and squamous cell carcinoma (OR 2.18, 95% CI 1.08–4.39, *p* = 0.028), prostate cancer (OR 2.85, 95% CI 1.09–7.54, *p* = 0.032), FDR history of melanoma (OR 2.37, 95% CI 1.31–4.29, *p* = 0.004), and FDR history of prostate cancer (OR 3.26, 95% CI 1.59–6.83, *p* = 0.001).

**Conclusions:**

Patients with MPM have a higher prevalence of personal and FDR histories of nonmelanoma skin cancers and prostate cancer.

## Graphical abstract

This retrospective case–control study enrolled patients (*n* = 378) with multiple primary melanoma (MPM) and single primary melanoma (SPM) at a 1:2 ratio and evaluated the association between MPM and other cancers using logistic regression models. Compared with patients with SPM, patients with MPM were more likely to have squamous cell carcinoma (OR 1.95, 95% CI 1.001–3.79, *p* = 0.047), basal cell carcinoma (OR 1.66, 95% CI 0.99–2.74, *p* = 0.051), and prostate cancer (OR 2.72, 95% CI 1.07–7.01, *p* = 0.034).

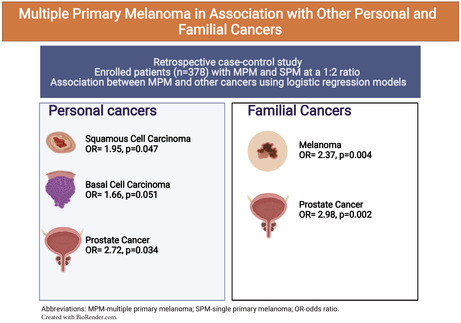

## 1 INTRODUCTION

The risk of nonmelanoma skin and nonskin cancers is increased in patients with melanoma, due either to shared environmental risk factors or the presence of mutations of certain cancer‐predisposing genes. UV exposure is the main cause of both melanoma and keratinocytic cancers, including basal cell carcinoma (BCC) and squamous cell carcinoma (SCC).^1^ Certain variants of melanocortin 1 receptor gene (MC1R), which encodes a protein that binds melanocyte‐stimulating hormone (MSH) and determines skin and hair pigmentation, confer risk for both melanoma and nonmelanoma skin cancers.^2^ Patients with familial atypical multiple mole melanoma (FAMMM), a hereditary cancer syndrome caused by mutation of cyclin‐dependent kinase inhibitor 2A (CDKN2A), are known to have a significantly higher risk of melanoma and pancreatic cancer.^3^ BRCA1‐associated protein 1 (BAP1) is another heritable cancer‐predisposing gene and its mutation has been reported to be related to mesothelioma, uveal melanoma, and cutaneous melanoma.^4^ Furthermore, patients with other inherited cancer syndromes, including Li Fraumeni syndrome, Cowden syndrome, and Hereditary Breast and Ovarian Cancer syndrome, have a poorly defined but elevated risk of melanoma.^5^ Despite many known shared risk factors, recent studies using statewide and nationwide cancer registries have revealed familial associations between melanoma and cancers that do not share the well‐established environmental factors or fall into typical predisposing cancer syndromes, including prostate cancer, colorectal cancer, nervous system cancer, and hematological malignancies.^6^, ^7^, ^8^ These emerging new links imply the presence of other shared environmental or genetic risk factors awaiting further investigation.

Patients with melanoma are at increased risk of developing subsequent melanomas.^9^, ^10^ Compared to patients with single primary melanoma (SPM), patients with MPM are more likely to have a younger age at onset, an earlier stage of melanoma (0 or I), and family history of melanoma.^10^, ^11^, ^12^, ^13^ Patients with FAMMM also present with multiple primary melanomas. However, whether the personal and familial cancer history differs between patients with MPM and SPM is not well known.

Here we conducted a retrospective case–control study to compare the personal cancer spectrum of these two groups. The secondary object of this study was to assess the association between MPM and the presence of other cancers among first‐degree relatives (FDRs).

## 2 MATERIALS AND METHODS

### 2.1 Study population and data source

This is a retrospective case–control study that enrolled gender‐matched patients with MPM as the study group and patients with SPM as the control group at a 1:2 ratio from the Melanoma Center of the UPMC Hillman Cancer Center. The diagnosis period of the study ranged from 1990 to 2019. Patients were seen and enrolled in the study from January 1996 to January 2020. This study is a part of the Human Biological Sample and Nevus Image Banking and Analysis protocol, which allows reviews of participants' medical records as well as retrieval of their preserved biological samples and digital imaging for research use. Cases with MPM were defined as those diagnosed with two or more pathology‐confirmed primary melanomas by the time of this study. Age at melanoma diagnosis, gender, melanoma stage, personal history of other cancers, cancer history of FDRs, and germline genetic mutation status were retrieved from the electronic medical record for review. Prostate cancer screening status, defined by the presence of serum Prostate‐Specific Antigen testing and/or prostate biopsy, was recorded for male patients. Age at diagnosis of prostate cancer, Gleason score, and metastatic status at last physician follow‐up was also obtained for male patients with melanoma and prostate cancer. This study was approved by the Institutional Review Board.

### 2.2 Statistical analysis

Categorical variables were presented as percentages and frequencies, and continuous variables were presented as medians and interquartile ranges (IQR). Comparisons of the variables were conducted between patients with SPM and with MPM. Kruskal–Wallis test was used for continuous variables. Univariate logistic regression models were used to determine the association between MPM and personal or family history of other cancers. Multivariable logistic regression models were adopted to assess the above associations after adjusting for age at the last physician follow‐up, gender, and initial melanoma stage with the exception that the association with a personal history of prostate cancer was adjusted for age and melanoma stage, but not gender. ORs and 95% confidence intervals (CIs) were reported. All tests were two‐sided. Statistical significance was defined as *p* < 0.05. Data were analyzed in R 4.1.0.

## 3 RESULTS

### 3.1 Characteristics of the whole cohort

In total, 378 melanoma patients were enrolled. The demographic information, personal cancer history, and cancer history of their first‐degree relatives (FDRs) are summarized in Table 1, Table S1, and Table S2. The median age of initial diagnosis of melanoma was 52 years old (IQR, 41–64 years old) and 167 (44.2%) patients were male. The majority (53%) of patients had early stage (0 or 1) melanoma at initial diagnosis. A total of 40 (10.6%) and 80 (21.2%) patients had a personal history of SCC or BCC, respectively, whereas 63 (16.7%) of patients had a personal history of noncutaneous cancers. The top 5 most common noncutaneous cancers of the whole cohort were prostate cancer (21, 12.6% in males), breast cancer (16, 7.6% in females), hematological malignancies (9, 2.4%), cervical cancer (4, 1.9% in females), and colon cancer (7, 1.9%). Less prevalent cancers were lung, renal cell carcinoma (RCC), sarcoma, thyroid, ovarian, uterine, neuroendocrine (NE), head and neck (HN), central nervous system (CNS), and bladder cancers (Table S1). Patients with FDR history of melanoma, nonmelanoma skin cancer, and noncutaneous cancers were 53 (14.0%), 32 (8.5%), and 179 (47.4%), respectively. The most common noncutaneous cancers in FDRs were breast cancer (45, 11.9%), prostate cancer (35, 9.3%), lung cancer (33, 8.3%), colon cancer (28, 7.4%), and hematological malignancies (24, 6.3%). Less prevalent noncutaneous cancers in FDR were RCC, HN, thyroid, esophageal, pancreas, bladder, CNS, gastric, uterine, cervical, bone, NE, and liver cancer (Table S2).

**TABLE 1:** Baseline characteristics of the patients (*n* = 378, male = 167, female = 211).

Variables	Value
Age, median (IQR), years	52 [41, 64]
Gender	
Male, no (%)	167 (44.2)
Female, no (%)	211 (55.8)
Staging, no (%)	
0	15 (3.9)
I	186 (49.2)
II	94 (25.1)
III	68 (18.2)
IV	11 (2.9)
PH of SCC, no (%)	40 (10.6)
PH of BCC, no (%)	80 (21.2)
PH of nonskin cancers, no (%)^a^	63 (16.7)
Prostate in males	21 (12.6)
Breast in females	16 (7.6)
Hematological malignancy	9 (2.4)
Cervical in females	4 (1.9)
Colorectal	7 (1.9)
Melanoma in FDR, no (%)	53 (14)
Nonmelanoma skin cancer in FDR, no (%)	32 (8.5)
Nonskin cancers in FDR, no (%)^b^	179 (47.4)
Breast	45 (11.9)
Prostate	35 (9.3)
Lung	33 (8.3)
Colorectal	28 (7.4)
Hematological malignancy	24 (6.3)

Abbreviations: BCC, basal cell carcinoma; FDR, first‐degree relative; IQR, interquartile range; PH, personal history; SCC, squamous cell carcinoma.

^a^ Only the top 5 most prevalent cancers are listed. Less prevalent cancers are listed in Table S1.

^b^ Only the top 5 most prevalent cancers are listed. Less prevalent cancers are listed in Table S2.

### 3.2 Basic characteristics of patients with SPM and MPM

In this cohort, patients with MPM and SPM were enrolled 1:2 (126 MPM, 252 SPM) and gender‐matched. The basic features of patients with MPM and SPM are summarized in Table 2. Compared to patients with SPM, patients with MPM were younger at the diagnosis of the first melanoma (median [IQR], 47 [34, 60] vs. 55 [44, 65], *p* = 0.001) and tended to have melanoma of earlier stage (0 or 1) (*p* < 0.001) and slightly but significantly longer follow‐up time (median [IQR], 9 years [6.00, 16.95] vs. 7 years [3.00, 10.00], *p* < 0.001). Among patients with MPM, the median time between the development of first primary melanoma and second primary melanoma was 19.5 months with IQR [2.0, 81.0], and 51.2% of patients were diagnosed with the second primary melanoma within the first 2 years of the diagnosis of first primary melanoma (Table S3). The second primary melanoma tended to be of the earlier stage than the first primary melanoma (*p* = 0.024) (Table S4).

**TABLE 2:** Basic features of patients with SPM and MPM.

Variables	MPM (*n* = 126)	SPM (*n* = 252)	*p* value
Age, median [IQR], years	47 [34.25, 60.75]	55 [44, 75.65]	0.001
Male, no (%)	53 (42.1)	114 (45.2)	0.634
Initial staging, no (%)			<0.001
0	14 (11.5)	1 (0.4)	
I	72 (59.0)	114 (45.6)	
II	23 (18.9)	71 (28.2)	
III	13 (10.7)	55 (21.8)	
IV	0 (0)	11 (4.4)	
Follow‐up, median [IQR], year	9 [6.00, 16.75]	7 [3.00, 10.00]	<0.001

Abbreviations: IQR, interquartile range; MPM, multiple primary melanomas; SPM, single primary melanoma.

### 3.3 Association between MPM and personal history of other cancers

Personal cancer histories are summarized in Table 3. The prevalence of SCC was marginally higher in patients with MPM (15.1% vs. 8.3%, OR 1.95, 95% CI 1.001–3.79, *p* = 0.047). There was also a trend toward a higher prevalence of BCC in patients with MPM compared with patients who had SPM, although this is not statistically significant (27% vs. 18.3%, OR 1.66, 95% CI 0.99–2.74, *p* = 0.051). No difference is seen in overall prevalence of nonskin cancers (16.7% vs. 16.7%, OR 1.12, 95% CI 0.63–1.94, *p* = 0.700). A significantly higher percentage of males with MPM had a history of prostate cancer than males with SPM (20.8% vs. 8.8%, OR 2.72, 95% CI 1.07–7.01, *p* = 0.034); prostate cancer screening rates are comparable between these two groups (54.7% vs. 49.1%, OR 1.25, 95% CI 0.65–2.42, *p* = 0.5). There is no difference among other noncutaneous cancers. We then performed a multivariable logistic regression analysis and found that the association remained significant between MPM and SCC (OR 2.18, 95% CI 1.08–4.39, *p* = 0.028) after adjusting for gender, age of the last visit, and initial melanoma stage (Table 5). The association between MPM and prostate cancer adjusted for age of last visit and initial melanoma stage remained significant (OR 2.85, 95% CI 1.09–7.54, *p* = 0.032) (Table 5). The prostate cancer screening rate was not included in the multivariable analysis due to the similar rates observed between the two groups.

**TABLE 3:** Personal history of cancer in patients with SPM and MPM.

Variables, no (%)	MPM	SPM	OR	95% CI	*p* Value
Sex‐unspecific cancers	*n* = 126	*n* = 252			
SCC	19 (15.1)	21 (8.3)	1.95	1.001, 3.79	0.047
BCC	34 (27)	46 (18.3)	1.66	0.99, 2.74	0.051
Nonskin cancers	21 (16.7)	42 (16.7)	1.12	0.63, 1.94	0.700
Hematological malignancy	2 (1.6)	7 (2.8)	0.56	0.08, 2.37	0.480
Colorectal	0 (0.0)	7 (2.8)	‐	‐	0.978
Lung	0 (0.0)	4 (1.6)	‐	‐	0.983
Renal cell carcinoma	0 (0.0)	2 (0.8)	‐	‐	0.982
Sarcoma	1 (0.8)	1 (0.4)	2.00	0.08, 51.06	0.620
Thyroid	0 (0.0)	2 (0.8)	‐	‐	0.982
Neuroendocrine	0 (0.0)	1 (0.4)	‐	‐	0.981
Head and Neck	1 (0.8)	0 (0.0)	‐	‐	0.978
CNS	1 (0.8)	0 (0.0)	‐	‐	0.978
Bladder	0 (0.0)	1 (0.4)	‐	‐	0.981
Male‐specific cancers	*n* = 53	*n* = 114			
Prostate cancer	11 (20.7)	10 (8.8%)	2.72	1.07, 7.01	0.034
Prostate cancer screening	29 (54.7)	56 (49.1)	1.25	0.65, 2.42	0.50
Female‐specific cancers	*n* = 73	*n* = 138			
Breast cancer	7 (9.6)	8 (5.8)	1.72	0.58, 5.00	0.312
Cervical cancer	0 (0.0)	4 (2.9)	‐	‐	0.980
Ovarian cancer	0 (0.0)	1 (0.7)	‐	‐	0.990
Uterine cancer	0 (0.0)	1 (0.7)	‐	‐	0.990

Abbreviations: 95% CI, 95% confidence interval; BCC, basal cell carcinoma; CNS, central nervous system; MPM, multiple primary melanomas; OR, odds ratio; SCC, squamous cell carcinoma; SPM, single primary melanoma.

### 3.4 Association between MPM and cancer history in FDRs

We then sought to determine the association between MPM and family cancer history (Table 4). Compared with patients who had an SPM, patients with MPM were more likely to have melanoma in FDRs (21.4% vs. 10.3%, OR 2.37, 95% CI 1.31–4.28, *p* = 0.004), but not nonmelanoma skin cancers (7.1% vs. 9.1%, OR 0.76, 95% CI 0.33–1.66, *p* = 0.515) or overall noncutaneous cancers (46.8% vs. 47.6%, OR 0.97, 95% CI 0.63–1.49, *p* = 0.884). Among cancer subtypes, prostate cancer was the most prevalent in FDRs of patients with MPM compared with patients who had SPM (15.9% vs. 6.0%, OR 2.98, 95% CI 1.47–6.14, *p* = 0.002). The prevalence of other noncutaneous cancers in FDRs did not vary between the two groups. The association between MPM and melanoma in FDRs (OR 2.37, 95% CI 1.31–4.29, *p* = 0.004), and prostate cancer in FDRs (OR 3.26, 95% CI 1.59–6.83, *p* = 0.001) remained significant after adjusting for gender, age of the last visit, and initial melanoma stage (Table 5).

**TABLE 4:** Cancer history in FDRs.

Variables, no (%)	MPM (*n* = 126)	SPM (*n* = 252)	OR	95% CI	*p* value
Melanoma	27 (21.4)	26 (10.3)	2.37	1.31, 4.28	0.004
Nonmelanoma skin cancer	9 (7.1)	23 (9.1)	0.76	0.33, 1.66	0.515
Nonskin cancers	59 (46.8)	120 (47.6)	0.97	0.63, 1.49	0.884
Breast	13 (10.3)	32 (12.7)	0.79	0.39, 1.53	0.501
Prostate	20 (15.9)	15 (6.0)	2.98	1.47, 6.14	0.002
Lung	6 (4.8)	27 (10.7)	0.42	0.15, 0.97	0.060
Colon	12 (9.5)	16 (6.3)	1.55	0.70, 3.38	0.270
Hematological malignancy	10 (7.9)	14 (5.6)	1.46	0.31, 3.35	0.373
Renal cell carcinoma	4 (3.2)	5 (2.0)	1.62	0.39, 6.23	0.478
Head and Neck	2 (1.6)	6 (2.4)	0.66	0.09, 2.92	0.616
Thyroid	3 (2.4)	4 (1.6)	1.51	0.29, 6.96	0.592
Esophageal	2 (1.6)	5 (2.0)	0.79	0.11, 3.75	0.788
Pancreatic	2 (1.6)	5 (2.0)	0.79	0.11, 3.75	0.788
Bladder	3 (2.4)	4 (1.6)	1.51	0.29, 6.96	0.592
CNS	1 (0.8)	4 (1.6)	0.49	0.03, 3.40	0.532
Gastric	1 (0.8)	4 (1.6)	0.49	0.03, 3.40	0.532
Uterine	0 (0.0)	5 (2.0)	‐	‐	0.982
Cervical	2 (1.6)	2 (0.8)	2.02	0.24, 16.96	0.486
Bone	1 (0.8)	3 (1.2)	0.66	0.03, 5.24	0.724
Neuroendocrine	0 (0.0)	2 (0.8)	‐	‐	0.982
Liver	1 (0.8)	1 (0.4)	2.01	0.08, 51.06	0.623

Abbreviations: 95% CI, 95% confidence interval; CNS, central nervous system; FDR: first‐degree relative; MPM, multiple primary melanomas; OR, odds ratio; SPM, single primary melanoma.

**TABLE 5:** Multivariable analysis between SPM and MPM.

MPM Yes/no	OR	95% CI	*p* Value
PH of SCC	2.18	1.08, 4.39	0.028
PH of prostate in males	2.85	1.09, 7.54	0.032
Melanoma in FDR	2.37	1.31, 4.29	0.004
Prostate Cancer in FDR	3.26	1.59, 6.83	0.001

Abbreviations: 95% CI, 95% confidence interval; FDR: first‐degree relative; MPM, multiple primary melanomas; OR, odds ratio; PH, personal history; SCC, squamous cell carcinoma; SPM, single primary melanoma.

### 3.5 Prostate cancer in melanoma patients

The possible familial association between melanoma and prostate cancer, suggested by the higher prevalence of prostate cancer in patients with MPM, prompted us to further explore the characteristics of prostate cancer among melanoma patients (Table 6). Of the 21 patients with prostate cancer, 11 had MPM while 10 had SPM. The overall median age of diagnosis of prostate cancer was 59 years old (IQR, 57.0–66.0 years old), which is younger than the reported 66 years age at the incidence of the general population.^14^ The median age of diagnosis was similar between patients with MPM and SPM. 60.0% of patients with SPM were diagnosed with prostate cancer prior to melanoma (median time from melanoma to prostate cancer diagnosis of −5.0 years [IQR, −8.7 to 0 years]), compared with 18.3% of patients with MPM (median time from the first primary and second primary melanoma to prostate cancer diagnosis of 2.0 years [IQR, 1.0–10.5 years], and 1.0 year [IQR, −9.5 to 2.5 years], respectively). The percentages who had Gleason scores of 6, 7, 8, and unknown were 27.3%, 54.5%, 0%, and 18.2%, respectively, among patients with MPM, and 10.0%, 30.0%, 20.0%, and 40.0%, respectively, among patients with SPM. None of the 21 patients had metastatic prostate cancer at initial diagnosis or last follow‐up.

**TABLE 6:** Characteristics of patients with both melanoma and prostate cancer (*n* = 21).

Variables	All (*n* = 21)	MPM (*n* = 11)	SPM (*n* = 10)
Age, median [IQR], years	59 [57.0, 66.0]	59.5 [57, 66.25]	60 [57.0, 67.0]
Diagnosis of prostate cancer, no (%)			
Prior to the first melanoma	8 (38.1)	2 (18.3)	6 (60.0)
Between first and second melanoma		3 (27.3)	
After second melanoma		6 (54.5)	
First melanoma to prostate cancer, median [IQR], years	0 [−8, 2]	2.0 [1.0, 10.5]	−5.0 [−8.7, 0]
Second melanoma to prostate cancer, median [IQR], years		1.0 [−9.5, 2.5]	
Gleason score, no (%)			
6	4 (19.0)	3 (27.3)	1 (10.0)
7	9 (42.9)	6 (54.5)	3 (30.0)
8	2 (9.5)	0 (0.0)	2 (20.0)
Unknown	6 (28.6)	2 (18.2)	4 (40.0)
Metastasis, no (%)	0 (0)	0 (0)	0 (0)
PH of third cancer, No (%)	11 (52.4)	5 (45.5)	6 (60.0)
BCC	7 (33.3)	5 (45.5)	3 (30.0)
SCC	4 (19.0)	3 (27.3)	1 (10.0)
Hematological malignancies	2 (9.5)	0 (0.0)	2 (20.0)
Neuroendocrine tumor	1 (4.8)	0 (0.0)	1 (10.0)
Other	0 (0)	0 (0.0)	0 (0.0)
Melanoma in FDR, No (%)	6 (28.6)	3 (27.3)	3 (30.0)
Nonmelanoma skin cancer in FDR, No (%)	2 (9.5)	0 (0.0)	2 (20.0)
Prostate cancer in FDR, no (%)	5 (23.8)	4 (36.4)	1 (10.0)
Other cancers in FDR, no (%)	14 (66.7)	10 (90.9)	4 (40.0)
Colorectal	4 (19.0)	3 (27.3)	1 (10.0)
Breast	3 (14.3)	2 (18.2)	1 (10.0)
Bladder	3 (14.3)	2 (18.2)	1 (10.0)
Hematological malignancies	2 (9.5)	2 (18.2)	0 (0.0)
Esophageal	1 (4.8)	1 (9.1)	0 (0.0)
Lung	1 (5.6)	1 (9.1)	0 (0.0)
Ovarian	1 (5.6)	1 (9.1)	0 (0.0)
CNS	1 (4.8)	0 (0.0)	1 (10.0)

Abbreviations: BCC, basal cell carcinoma; CNS, central nervous system; FDR, first‐degree relative; IQR, interquartile ranges; MPM, multiple primary melanomas; PH, personal history; SCC, squamous cell carcinoma; SPM, single primary melanoma.

Among the 21 patients, 11 (52.4%) patients had a third cancer, including BCC (33.3%), SCC (19.0%), hematological malignancies (9.5%), and colon cancer (4.8%). No other types of cancer were observed, although the total number of patients is small. Regarding family history, 23.8% were positive for prostate cancer in FDRs. Patients with an FDR history of melanoma, nonmelanoma skin cancer, and other noncutaneous cancer were 28.6%, 9.5%, and 66.7%, respectively. The most common noncutaneous nonprostate cancers in FDRs were colon cancer (19%), breast cancer (14.3%), and bladder cancer (14.3%). Less prevalent cancers in FDR were hematological malignancies, esophageal, lung, ovarian, and CNS cancers (Table 6). Due to the sample size, the personal and family cancer history of patients with MPM and SPM were descriptively outlined (Table 6) without statistical comparison. There were trends toward a higher prevalence of BCC (45.5% vs. 30.0%), SCC (27.3% vs. 10.0%), family history of prostate cancer (36.4% vs. 10.0%), and family history of colorectal cancer (27.3% vs. 10.0%) among patients with MPM and prostate cancer compared with patients who had SPM and prostate cancer; however, the larger sample size is required for validation.

Next, we sought to determine whether there were potential underlying predisposing germline gene mutations shared by these two cancers. Of the 21 patients, only 1 patient received the germline gene mutation test. This patient had MPM, prostate cancer, BCC, and a family history of melanoma and lung cancer. He was found to have a single‐nucleotide variant (c.1572G > T) of MutL homolog 1 (MLH1) (data not shown), a mismatch repair gene already associated with hereditary nonpolyposis colorectal cancer (HNPCC), or Lynch syndrome. However, this substitution mutation is of uncertain significance. Other genes tested included ATM, BAP1, BRCA1, BRCA2, BRIP1, CDK4, CDKN2A p14ARF, CDK2A p161NK4a, CHEK2, EPCAM, FANCA, MC1R, MSH2, MSH6, NBN, PALB2, PMS2, POT1, PTEN, RAD51C, RAD51D, RB1, TERT, and TP53. All of these proved to be negative for mutations.

## 4 DISCUSSION

Personal and familial cancer history of patients with SPM and MPM has not directly been evaluated or compared in the past. In this single‐center, retrospective case–control study, the cancer history of the two populations and their FDRs were thoroughly reviewed and compared. Our study shows a possible familial association between MPM and keratinocytic cancers and prostate cancers.

We first delineated the cancer history of the whole cohort and their FDRs. The prevalence of SCC and BCC was 10.6% and 21.2%, respectively. The top 5 noncutaneous cancers in the cohort were prostate cancer, breast cancer, hematological malignancies, cervical cancers, and colon cancers. This finding aligns with the cancer distribution of the general population except that cervical cancer ranked higher in our cohort, perhaps resulting from better adherence to cancer screening. As for family cancer history, the prevalence of melanoma and the distribution of noncutaneous cancers in FDRs were both comparable to that of the general population.^14^

We then carried out a comparison between patients with SPM and MPM. Interestingly, our study showed that the prevalence of prostate cancer was increased in the MPM group and their FDRs, in both univariate and multivariable regression analyses, suggesting a potential familial association between melanoma and prostate cancer. Emerging evidence from other studies also indicates the possible association between these two cancers. Frank et al., using a nationwide cancer database of the Sweden population, showed a relative risk (RR) of 1.17 (95% CI 1.11–2.64) for prostate cancer when two or more FDRs had melanoma, with at least one FDR having MPM.^6^ A large prospective cohort study of males in the United States demonstrated an increased incidence of melanoma among patients with a personal history of prostate cancer (multivariable‐adjusted hazard ratio 1.83, 95% CI 1.32–2.54).^15^ Moreover, an analysis of a statewide cancer registry in Utah revealed a bidirectional association between melanoma and prostate cancer, where RR was 1.26 (95% CI 1.07–1.63) for prostate cancer among FDRs of melanoma patients, and 1.16 (95% CI 1.09–1.23) for melanoma among FDRs of prostate cancer patients.^7^

Despite these findings, whether a real association exists between melanoma and prostate cancers remains controversial. Another nationwide cohort from Sweden reported an increased risk of melanoma in men with prostate cancer (multivariable‐adjusted hazard ratio 1.18, 95% CI 1.09–1.27), however, the risk was only seen for early stage melanoma in men with low‐risk prostate cancer and high educational and economic status.^16^ Therefore, the observed association was thought to be the potential consequence of well‐educated, wealthy men being more likely to spend holidays on a sunny beach, leading to increased melanoma risk, and to receipt of health checks that included skin examination and prostate cancer screening.^17^

Given that the same concern also applies to the comparison between patients with MPM and SPM, we included a notation of prostate cancer screening status in our study. The two groups had a comparably high rate of screening, indicating that the higher prevalence of prostate cancer in patients with MPM was not likely to be the result of higher rates of prostate cancer screening. No previous head‐to‐head comparison of the prevalence of prostate cancer has been conducted between patients with SPM and MPM. Given the limited sample size of our cohort, the increased risk of prostate cancer in patients with MPM needs further validation.

In a review of the underlying linkage between melanoma and prostate cancer, we then explored whether germline mutation was present in patients with both cancers. More interestingly, 5 out of 11 (45.5%) patients with MPM and prostate cancer also had BCC, which further raised the question of whether these three cancers had shared predisposing gene mutation. Unfortunately, only 1 out of 21 patients who had MPM, prostate cancer, and BCC, underwent germline genetic testing. This patient was found to have c.1572G > T (p. Met524Ile) of MLH1, a missense variant whose presence was previously reported in ovarian cancer patients although its role in cancer pathogenesis has been uncertain.^18^ Although neither BCC, prostate cancer, or melanoma falls into the typical cancer clusters of Lynch syndrome, certain germline MLH1 variants, not including the one our patient carries, have been reported to be associated with familial melanoma and familial prostate cancer.^5^, ^19^ Besides MLH1, men carrying mutations of other DNA mismatch repair (MMR) genes, including MSH2, MSH6, and PMS2, were also found to have an increased risk of prostate cancer (RR 3.67, 95% CI 2.32–6.67) in a meta‐analysis.^20^ Also, high prevalence and earlier onset of melanoma were reported among patients with Lynch syndrome.^21^ Few data exist on the correlation between MMR genes and BCC, but certain single‐nucleotide polymorphisms (SNP) of MLH1 were found to increase the risk of BCC development in the Brazilian population.^22^ Thus, additional cancer clusters may exist in the MMR gene mutation carriers beyond the traditional cancer spectrum of Lynch syndrome, and further investigation is needed to validate the hypothesis.

Recent studies also reveal other shared predisposing gene mutations among melanoma, prostate cancer, and potentially, BCC. For example, loss‐of‐function (LOF) variants and variants of unknown significance (VUS) in the ataxia‐telangiectasia‐mutated (ATM) gene were reported to be enriched in cases with MPM, and the most common nonmelanoma cancers among these patients were BCC followed by prostate cancer.^23^ Mutations of BRCA1 and BRCA2 were well known as a risk factor for hereditary breast cancer and there were significantly increased risks of melanoma and prostate cancer in BRCA mutation carriers.^24^, ^25^, ^26^, ^27^, ^28^ Limited data were available on BRCA and BCC. One study showed that BRCA2 mutation carriers were more likely to develop BCC compared with BRCA1 mutation carriers,^29^ however, the incidence of BCC was not compared between BRCA mutation carriers and the general population. In addition, mutations of BRIP1, PTEN, TP53, and TERT were all reported to associate with an increased risk of melanoma and prostate cancer.^25^, ^30^, ^31^, ^32^ More robust genetic testing in patients with both melanoma and prostate cancer will help further establish the molecular linkage between these two cancers.

The strengths of this study are the thorough data collection including detailed melanoma history, personal history of nonmelanoma cancers, cancer history of FDRs, genetic testing results, cancer screening information, and basic history of prostate cancer. The availability of prostate cancer screening status enabled us to minimize its confounding effect on prostate cancer diagnosis and demonstrate a more solid association between melanoma and prostate cancer. This study has several limitations. First, it is retrospective and from a single center. The findings need to be validated before application to more general populations. Second, recall bias might exist in the cancer history of FDRs as histories were self‐reported. Third, the exact number of first‐ and second‐degree relatives of patients with SPM and MPM and the cancer history of each relative were unavailable for review. Fourth, the size of the patient sample with prostate cancer was too small to perform statistical analysis for comparison of the feature of prostate cancer between SPM and MPM. Finally, we lack robust genetic testing to establish the underlying genetic linkage between melanoma and prostate cancer. Nevertheless, our study is the first to compare the cancer history of patients with SPM and MPM head‐to‐head, and we have found a potential linkage between MPM and prostate cancer, which will serve as a foundation for future epidemiological and genetic studies between these two cancers.

In conclusion, patients with MPM have a similar personal and family cancer history spectrum compared to patients with SPM, except for the higher prevalence of prostate cancer, without any significant disparity in prostate cancer screening rates of these two populations. In addition, familial associations may exist between melanoma and prostate cancer as a result of shared inherited gene mutations that warrant further investigation and validation.

## AUTHOR CONTRIBUTIONS

Acquisition of data: XY, LK, AK, CS, SR, CM, and MW. Analysis and interpretation of data: XY, ZH, LK, and JMK. Drafting of the manuscript: All authors. Critical revision: XY, LK, ZH, and JMK.

## FUNDING INFORMATION

This work was supported by the Melanoma Center Internal Funding.

## CONFLICT OF INTEREST

XY, LK, ZH, AK, SR, CS, CM, and MW declare no conflict of interest. JMK Consultant: Amgen, Inc, Ankyra Therapeutics, Axio Research/Instil Bio, BMS, Checkmate Pharmaceuticals, DermTech, Elsevier, Harbour BioMed, Immunocore LLC, Iovance Biotherapeutics, Istari Oncology, Millennium Pharmaceuticals/Takeda Pharmaceutical, Natera Inc., Novartis Pharmaceuticals, OncoCyte Corporation, OncoSec Medical Inc., Pfizer, Scopus BioPharma, Replimune. Research support: BMS, Amgen, Inc, Checkmate, Castle Biosciences, Inc., Immunocore LLC, Iovance, Novartis, Merck.

## ETHICS STATEMENT

This study was approved by the Institutional Review Board of the University of Pittsburgh.

## Supporting information


Table S1
Table S2
Table S3
Table S4

## Data Availability

The data that support the findings of this study are available from the corresponding author upon reasonable request.
