# Supplementary material for: Multiple primary melanoma in association with other personal and familial cancers
Source: Cancer Med. 2022 Aug 5;12(3):2474–83. doi: 10.1002/cam4.5088 (PMC9939182; doi:10.1002/cam4.5088)
Supplement: Supplementary file 1 — Table S1 Table S2 Table S3 Table S4 [file CAM4-12-2474-s001.docx]

**Table 1. Baseline Characteristics of the Patients**

**(n=378, male=167, female = 211)**

| Variables | Value |
| --- | --- |
| Age, median (IQR), y | 52 [41, 64] |
| Gender |  |
| Male, No (%) | 167 (44.2) |
| Female, No (%) | 211 (55.8) |
| Staging, No (%) |  |
| 0 | 15 (3.9) |
| I | 186 (49.2) |
| II | 94 (25.1) |
| III | 68 (18.2) |
| IV | 11 (2.9) |
| PH of SCC, No (%) | 40 (10.6) |
| PH of BCC, No (%) | 80 (21.2) |
| PH of non-skin cancers, No (%)^a^ | 63 (16.7) |
| Prostate in males | 21 (12.6) |
| Breast in females | 16 (7.6) |
| Hematological malignancy | 9 (2.4) |
| Cervical in females | 4 (1.9) |
| Colorectal | 7 (1.9) |
| Melanoma in FDR, No (%) | 53 (14) |
| Non-melanoma skin cancer in FDR, No (%) | 32 (8.5) |
| Non-skin cancers in FDR, No (%)^b^ | 179 (47.4) |
| Breast | 45 (11.9) |
| Prostate | 35 (9.3) |
| Lung | 33 (8.3) |
| Colorectal | 28 (7.4) |
| Hematological malignancy | 24 (6.3) |

a: Only top 5 most prevalent cancers are listed. Less prevalent cancers are listed in Table S1.

b: Only top 5 most prevalent cancers are listed. Less prevalent cancers are listed in Table S2.

c. Abbreviation: IQR, interquartile ranges; PH, personal history; SCC, squamous cell carcinoma; BCC: basal cell carcinoma; FDR: first-degree relative.

**Table 2. Basic Features of Patients with SPM and MPM**

| Variables | MPM (n=126) | SPM (n=252) | p Value |
| --- | --- | --- | --- |
| Age, median [IQR], y | 47 [34.25, 60.75] | 55 [44, 75.65] | 0.001 |
| Male, No (%) | 53 (42.1) | 114 (45.2) | 0.634 |
| Initial Staging, No (%) |  |  | <0.001 |
| 0 | 14 (11.5) | 1 (0.4) |  |
| I | 72 (59.0) | 114 (45.6) |  |
| II | 23 (18.9) | 71 (28.2) |  |
| III | 13 (10.7) | 55 (21.8) |  |
| IV | 0 (0) | 11 (4.4) |  |
| Follow-up, median [IQR], y | 9 [6.00, 16.75] | 7 [3.00, 10.00] | <0.001 |

Abbreviation: SPM, single primary melanoma; MPM, multiple primary melanomas; IQR, interquartile ranges.

**Table 3. Personal History of Cancer in Patients with SPM and MPM**

| Variables, No (%) | MPM | SPM | OR | 95% CI | p Value |
| --- | --- | --- | --- | --- | --- |
| Sex-unspecific cancers | n=126 | n=252 |  |  |  |
| SCC | 19 (15.1) | 21 (8.3) | 1.95 | 1.001, 3.79 | 0.047 |
| BCC | 34 (27) | 46 (18.3) | 1.66 | 0.99, 2.74 | 0.051 |
| non-skin cancers | 21 (16.7) | 42 (16.7) | 1.12 | 0.63, 1.94 | 0.700 |
| Hematological malignancy | 2 (1.6) | 7 (2.8) | 0.56 | 0.08, 2.37 | 0.480 |
| Colorectal | 0 (0.0) | 7 (2.8) | - | - | 0.978 |
| Lung | 0 (0.0) | 4 (1.6) | - | - | 0.983 |
| Renal cell carcinoma | 0 (0.0) | 2 (0.8) | - | - | 0.982 |
| Sarcoma | 1 (0.8) | 1 (0.4) | 2.00 | 0.08, 51.06 | 0.620 |
| Thyroid | 0 (0.0) | 2 (0.8) | - | - | 0.982 |
| Neuroendocrine | 0 (0.0) | 1 (0.4) | - | - | 0.981 |
| Head and Neck | 1 (0.8) | 0 (0.0) | - | - | 0.978 |
| CNS | 1 (0.8) | 0 (0.0) | - | - | 0.978 |
| Bladder | 0 (0.0) | 1 (0.4) | - | - | 0.981 |
| Male-specific cancers | n=53 | n=114 |  |  |  |
| Prostate cancer | 11 (20.7) | 10 (8.8%) | 2.72 | 1.07, 7.01 | 0.034 |
| Prostate cancer screening | 29 (54.7) | 56 (49.1) | 1.25 | 0.65, 2.42 | 0.50 |
| Female-specific cancers | n=73 | n=138 |  |  |  |
| Breast cancer | 7 (9.6) | 8 (5.8) | 1.72 | 0.58, 5.00 | 0.312 |
| Cervical cancer | 0 (0.0) | 4 (2.9) | - | - | 0.980 |
| Ovarian cancer | 0 (0.0) | 1 (0.7) | - | - | 0.990 |
| Uterine cancer | 0 (0.0) | 1 (0.7) | - | - | 0.990 |

Abbreviation: SPM, single primary melanoma; MPM, multiple primary melanomas; BCC, basal cell carcinoma; SCC, squamous cell carcinoma; CNS, central nervous system; OR, odds ratio; 95%CI, 95% confidence intervals.

**Table 4. Cancer History in FDRs**

| Variables, No (%) | MPM (n=126) | SPM (n=252) | OR | 95% CI | p Value |
| --- | --- | --- | --- | --- | --- |
| Melanoma | 27 (21.4) | 26 (10.3) | 2.37 | 1.31, 4.28 | 0.004 |
| Non-melanoma skin cancer | 9 (7.1) | 23 (9.1) | 0.76 | 0.33, 1.66 | 0.515 |
| Non-skin cancers | 59 (46.8) | 120 (47.6) | 0.97 | 0.63, 1.49 | 0.884 |
| Breast | 13 (10.3) | 32 (12.7) | 0.79 | 0.39, 1.53 | 0.501 |
| Prostate | 20 (15.9) | 15 (6.0) | 2.98 | 1.47, 6.14 | 0.002 |
| Lung | 6 (4.8) | 27 (10.7) | 0.42 | 0.15, 0.97 | 0.060 |
| Colon | 12 (9.5) | 16 (6.3) | 1.55 | 0.70, 3.38 | 0.270 |
| Hematological malignancy | 10 (7.9) | 14 (5.6) | 1.46 | 0.31, 3.35 | 0.373 |
| Renal cell carcinoma | 4 (3.2) | 5 (2.0) | 1.62 | 0.39, 6.23 | 0.478 |
| Head and Neck | 2 (1.6) | 6 (2.4) | 0.66 | 0.09, 2.92 | 0.616 |
| Thyroid | 3 (2.4) | 4 (1.6) | 1.51 | 0.29, 6.96 | 0.592 |
| Esophageal | 2 (1.6) | 5 (2.0) | 0.79 | 0.11, 3.75 | 0.788 |
| Pancreatic | 2 (1.6) | 5 (2.0) | 0.79 | 0.11, 3.75 | 0.788 |
| Bladder | 3 (2.4) | 4 (1.6) | 1.51 | 0.29, 6.96 | 0.592 |
| CNS | 1 (0.8) | 4 (1.6) | 0.49 | 0.03, 3.40 | 0.532 |
| Gastric | 1 (0.8) | 4 (1.6) | 0.49 | 0.03, 3.40 | 0.532 |
| Uterine | 0 (0.0) | 5 (2.0) | - | - | 0.982 |
| Cervical | 2 (1.6) | 2 (0.8) | 2.02 | 0.24, 16.96 | 0.486 |
| Bone | 1 (0.8) | 3 (1.2) | 0.66 | 0.03, 5.24 | 0.724 |
| Neuroendocrine | 0 (0.0) | 2 (0.8) | - | - | 0.982 |
| Liver | 1 (0.8) | 1 (0.4) | 2.01 | 0.08, 51.06 | 0.623 |

Abbreviation: SPM, single primary melanoma; MPM, multiple primary melanomas; CNS, central nervous system; OR, odds ratio; 95%CI, 95% confidence intervals.

**Table 5. Multivariable Analysis Between SPM and MPM**

| MPM Yes/no | OR | 95% CI | p Value |
| --- | --- | --- | --- |
| PH of SCC | 2.18 | 1.08, 4.39 | 0.028 |
| PH of prostate in males | 2.85 | 1.09, 7.54 | 0.032 |
| Melanoma in FDR | 2.37 | 1.31, 4.29 | 0.004 |
| Prostate Cancer in FDR | 3.26 | 1.59, 6.83 | 0.001 |

Abbreviation: MPM, multiple primary melanomas; PH: personal history; SCC, squamous cell carcinoma; FDR: first-degree relative; OR, odds ratio; 95%CI, 95% confidence intervals.

**Table 6. Characteristics of patients with both melanoma and prostate cancer (n=21)**

| Variables | All (n=21) | MPM (n=11) | SPM (n=10) |
| --- | --- | --- | --- |
| Age, median [IQR], y | 59 [57.0, 66.0] | 59.5 [57, 66.25] | 60 [57.0, 67.0] |
| Diagnosis of prostate cancer, No (%) |  |  |  |
| Prior to 1st melanoma | 8 (38.1) | 2 (18.3) | 6 (60.0) |
| Between 1st and 2nd melanoma |  | 3 (27.3) |  |
| After 2^nd^ melanoma |  | 6 (54.5) |  |
| 1^st^ melanoma to prostate cancer, median [IQR], y | 0 [-8, 2] | 2.0 [1.0, 10.5] | -5.0 [-8.7, 0] |
| 2^nd^ melanoma to prostate cancer, median [IQR], y |  | 1.0 [-9.5, 2.5] |  |
| Gleason score, No (%) |  |  |  |
| 6 | 4 (19.0) | 3 (27.3) | 1 (10.0) |
| 7 | 9 (42.9) | 6 (54.5) | 3 (30.0) |
| 8 | 2 (9.5) | 0 (0.0) | 2 (20.0) |
| unknown | 6 (28.6) | 2 (18.2) | 4 (40.0) |
| Metastasis, No (%) | 0 (0) | 0 (0) | 0 (0) |
| PH of a 3^rd^ cancer, No (%) | 11 (52.4) | 5 (45.5) | 6 (60.0) |
| BCC | 7 (33.3) | 5 (45.5) | 3 (30.0) |
| SCC | 4 (19.0) | 3 (27.3) | 1 (10.0) |
| Hematological malignancies | 2 (9.5) | 0 (0.0) | 2 (20.0) |
| Neuroendocrine tumor | 1 (4.8) | 0 (0.0) | 1 (10.0) |
| Other | 0 (0) | 0 (0.0) | 0 (0.0) |
| Melanoma in FDR, No (%) | 6 (28.6) | 3 (27.3) | 3 (30.0) |
| Non-melanoma skin cancer in FDR, No (%) | 2 (9.5) | 0 (0.0) | 2 (20.0) |
| Prostate cancer in FDR, No (%) | 5 (23.8) | 4 (36.4) | 1 (10.0) |
| Other cancers in FDR, No (%) | 14 (66.7) | 10 (90.9) | 4 (40.0) |
| Colorectal | 4 (19.0) | 3 (27.3) | 1 (10.0) |
| Breast | 3 (14.3) | 2 (18.2) | 1 (10.0) |
| Bladder | 3 (14.3) | 2 (18.2) | 1 (10.0) |
| Hematological malignancies | 2 (9.5) | 2 (18.2) | 0 (0.0) |
| Esophageal | 1 (4.8) | 1 (9.1) | 0 (0.0) |
| Lung | 1 (5.6) | 1 (9.1) | 0 (0.0) |
| Ovarian | 1 (5.6) | 1 (9.1) | 0 (0.0) |
| CNS | 1 (4.8) | 0 (0.0) | 1 (10.0) |

Abbreviation: SPM, single primary melanoma; MPM, multiple primary melanomas; FDR, first-degree relative; BCC, basal cell carcinoma; SCC, squamous cell carcinoma; IQR, interquartile ranges; PH, personal history

**Supplementary Tables**

**Table S1. Personal History of Non-Skin Cancers**

**(n=378, male=167, female = 211)**

| Variables | Value, No (%) |
| --- | --- |
| Prostate in males | 18 (10.8) |
| Breast in females | 16 (7.6) |
| Hematological malignancy | 9 (2.4) |
| Cervical in females | 4 (1.9) |
| Colorectal | 7 (1.9) |
| Lung | 4 (1.1) |
| Renal cell carcinoma | 2 (0.5) |
| Sarcoma | 2 (0.5) |
| Thyroid | 2 (0.5) |
| Ovarian in females | 1 (0.5) |
| Uterine in females | 1 (0.5) |
| Neuroendocrine | 1 (0.3) |
| Head and Neck | 1 (0.3) |
| CNS | 1 (0.3) |
| Bladder | 1 (0.3) |

**Table S2. History of Non-Skin Cancers in FDR**

| Variables | Value, No (%) |
| --- | --- |
| Breast | 45 (11.9) |
| Prostate | 35 (9.3) |
| Lung | 33 (8.3) |
| Colorectal | 28 (7.4) |
| Hematological malignancy | 24 (6.3) |
| Renal cell carcinoma | 9 (2.4) |
| Head and Neck | 8 (2.1) |
| Thyroid | 7 (1.9) |
| Esophageal | 7 (1.9) |
| Pancreatic | 7 (1.9) |
| Bladder | 7 (1.9) |
| CNS | 5 (1.3) |
| Gastric | 5 (1.3) |
| Uterine | 5 (1.3) |
| Cervical | 4 (1.1) |
| Bone | 4 (1.1) |
| Neuroendocrine | 2 (0.5) |
| Liver | 2 (0.5) |

**Table S3. Time between first primary melanoma and second primary melanoma in MPM patients**

| Median [IQR], months | 19.5 [2.0, 81.0] |
| --- | --- |
| Synchronized, n(%) | 29 (20.3) |
| <1 year | 24 (19.0) |
| 1-2 years | 15(11.9) |
| 2-5 years | 23 (18.3) |
| 5-10 years | 13 (10.3) |
| >10 years | 22 (17.5) |

**Table S4. Staging of first primary melanoma and second primary melanoma in MPM patients**

| Staging | First primary | Second primary | p Value |
| --- | --- | --- | --- |
| 0 | 14 (11.5) | 28 (22.8) |  |
| I | 72 (59.0) | 73 (61.0) | 0.024 |
| II | 23 (18.9) | 14 (11.4) |  |
| III | 13 (10.7) | 6 (4.9) |  |
| IV | 0 (0) | 0 (0.0) |  |
